# Human adipose tissue mesenchymal stromal cells and their extracellular vesicles act differentially on lung mechanics and inflammation in experimental allergic asthma

**DOI:** 10.1186/s13287-017-0600-8

**Published:** 2017-06-24

**Authors:** Ligia Lins de Castro, Debora Gonçalves Xisto, Jamil Zola Kitoko, Fernanda Ferreira Cruz, Priscilla Christina Olsen, Patricia Albuquerque Garcia Redondo, Tatiana Paula Teixeira Ferreira, Daniel Jay Weiss, Marco Aurélio Martins, Marcelo Marcos Morales, Patricia Rieken Macedo Rocco

**Affiliations:** 1 Laboratory of Pulmonary Investigation, Carlos Chagas Filho Biophysics Institute, Federal University of Rio de Janeiro, Avenida Carlos Chagas Filho, 373, Bloco G-014, Ilha do Fundão, 21941-902 Rio de Janeiro, RJ Brazil; 2 Laboratory of Cellular and Molecular Physiology, Carlos Chagas Filho Biophysics Institute, Federal University of Rio de Janeiro, Rio de Janeiro, RJ Brazil; 3 Laboratory of Clinical Bacteriology and Immunology, Health Sciences Center, Federal University of Rio de Janeiro, Rio de Janeiro, RJ Brazil; 4 Program of Morphological Sciences, Biomedical Sciences Institute, Federal University of Rio de Janeiro, Rio de Janeiro, RJ Brazil; 5 Laboratory of Inflammation, Oswaldo Cruz Institute, Oswaldo Cruz Foundation, Rio de Janeiro, RJ Brazil; 6 Department of Medicine, University of Vermont, College of Medicine, Burlington, VT USA

**Keywords:** Asthma, Remodeling, Mesenchymal stromal cells, Extracellular vesicles, Inflammation

## Abstract

**Background:**

Asthma is a chronic inflammatory disease that can be difficult to treat due to its complex pathophysiology. Most current drugs focus on controlling the inflammatory process, but are unable to revert the changes of tissue remodeling. Human mesenchymal stromal cells (MSCs) are effective at reducing inflammation and tissue remodeling; nevertheless, no study has evaluated the therapeutic effects of extracellular vesicles (EVs) obtained from human adipose tissue-derived MSCs (AD-MSC) on established airway remodeling in experimental allergic asthma.

**Methods:**

C57BL/6 female mice were sensitized and challenged with ovalbumin (OVA). Control (CTRL) animals received saline solution using the same protocol. One day after the last challenge, each group received saline, 10^5^ human AD-MSCs, or EVs (released by 10^5^ AD-MSCs). Seven days after treatment, animals were anesthetized for lung function assessment and subsequently euthanized. Bronchoalveolar lavage fluid (BALF), lungs, thymus, and mediastinal lymph nodes were harvested for analysis of inflammation. Collagen fiber content of airways and lung parenchyma were also evaluated.

**Results:**

In OVA animals, AD-MSCs and EVs acted differently on static lung elastance and on BALF regulatory T cells, CD3^+^CD4^+^ T cells, and pro-inflammatory mediators (interleukin [IL]-4, IL-5, IL-13, and eotaxin), but similarly reduced eosinophils in lung tissue, collagen fiber content in airways and lung parenchyma, levels of transforming growth factor-β in lung tissue, and CD3^+^CD4^+^ T cell counts in the thymus. No significant changes were observed in total cell count or percentage of CD3^+^CD4^+^ T cells in the mediastinal lymph nodes.

**Conclusions:**

In this immunocompetent mouse model of allergic asthma, human AD-MSCs and EVs effectively reduced eosinophil counts in lung tissue and BALF and modulated airway remodeling, but their effects on T cells differed in lung and thymus. EVs may hold promise for asthma; however, further studies are required to elucidate the different mechanisms of action of AD-MSCs versus their EVs.

## Background

Most patients with asthma achieve disease control with a combination of corticosteroids and long-acting β_2_-adrenoceptor agonists [1]. These drugs minimize inflammation and slow accompanying airway remodeling. However, as they are unable to revert established remodeling [2, 3], many patients still experience severe exacerbations or unsuccessful control of their symptoms [4, 5].

An ideal therapy for asthma would effectively act not only on inflammation but also on airway remodeling. Systemic administration of adipose tissue-derived mesenchymal stromal cells (AD-MSCs) is able to both improve lung function and reduce inflammation in ovalbumin (OVA)-induced asthma [6–9]. In occupational asthma induced by ammonium persulfate in mice, Martinez-Gonzalez et al. [10] demonstrated that AD-MSCs have potential anti-inflammatory effects and reduce smooth muscle hypertrophy and vascular hyperemia. In a mouse model of severe asthma induced by *Aspergillus* hyphal extract, systemically administered extracellular vesicles (EVs) derived from human bone marrow-derived MSCs were equally effective as the MSCs themselves in ameliorating airway hyperresponsiveness, histologic inflammation, and inflammatory markers in bronchoalveolar lavage fluid (BALF); however, airway remodeling was not specifically evaluated [11]. Further, to date, no study has evaluated the therapeutic potential of EVs derived from any source of MSCs once airway remodeling is already established. Within this context, the present study comparatively assessed effects of systemic administration of human AD-MSCs versus EVs derived from the AD-MSCs in a model of severe ovalbumin-induced allergic inflammation and airway remodeling in immunocompetent mice.

## Methods

This study was approved by the Ethics Committee of the Federal University of Rio de Janeiro Health Sciences Center (CEUA-UFRJ: 007/14, Rio de Janeiro, Brazil). All animals received humane care by trained veterinarians and veterinary staff in compliance with the “Principles of Laboratory Animal Care” formulated by the National Society for Medical Research and the *Guide for the Care and Use of Laboratory Animals* prepared by the U.S. National Academy of Sciences.

### Animal preparation

Sixty-eight female C57BL/6 mice (weight 19–21 g, age 2–3 months) were used. As bronchoalveolar lavage may affect lung morphological analysis and compromise lung function, 28 female C57BL/6 mice were used to evaluate lung mechanics and histology as well as levels of cytokines and growth factors in lung tissue (n = 7/group), and another 40 female animals were used to analyze total and differential cell counts in BALF, lymph nodes, and thymus (n = 10/group).

### AD-MSC isolation and culture conditions

With Institutional Ethics Committee approval (CEP-UFRJ: 088/04, Rio de Janeiro, Brazil), human adipose tissue was obtained from healthy adult women (aged 21 to 45 years) undergoing elective abdominal plastic surgery at Hospital Clementino Fraga Filho, Federal University of Rio de Janeiro. Adipose tissues were enzymatically digested using 0.1% type I collagenase (Sigma-Aldrich, St. Louis, MO, USA) at 37 °C for 30 minutes, under agitation. The cell pellet obtained was filtered in nylon membranes (10 mm), and then centrifuged twice at 230 × *g* for 5 minutes. After isolation, 10^6^ adipose tissue-derived cells were cultured (37 ° C, 5% CO_2_) in Dulbecco’s modified Eagle’s medium (DMEM) (Life Technologies, Grand Island, NY, USA) high-glucose medium containing 20% fetal bovine serum (FBS, Life Technologies, Grand Island, NY, USA), 100 units/mL penicillin, and 100 μg/mL streptomycin antibiotic solution (Gibco, Albuquerque, NM, USA). Twenty-four hours after initial culture, the medium was replaced, removing nonadherent cells. Adherent cells at approximately 80% confluence were detached from the cell culture flask using 0.05% trypsin-EDTA (Gibco, Albuquerque, NM, USA) and replated. These cells have been extensively characterized for cell surface marker expression and differentiation capacity [12]. For use in experiments, cell viability, density, and final concentration (1 × 10^5^ viable cells per 200 μl of phosphate-buffered saline [PBS]) was determined by trypan blue exclusion and by counting in a hemocytometer [11].

### EV extraction and characterization

To ensure a massive release of EVs by AD-MSC, stress induction of these cells was achieved through full depletion of FBS contained in the culture medium for 12 hours [13]. The medium was collected and centrifuged at 2000 × *g* for 20 minutes at 4 °C to remove cellular debris, followed by ultracentrifugation (100,000 × *g*) for 2 hours at 4 °C. The precipitate was collected and suspended in 0.9% saline solution for immediate use.

**Fig. 1:**
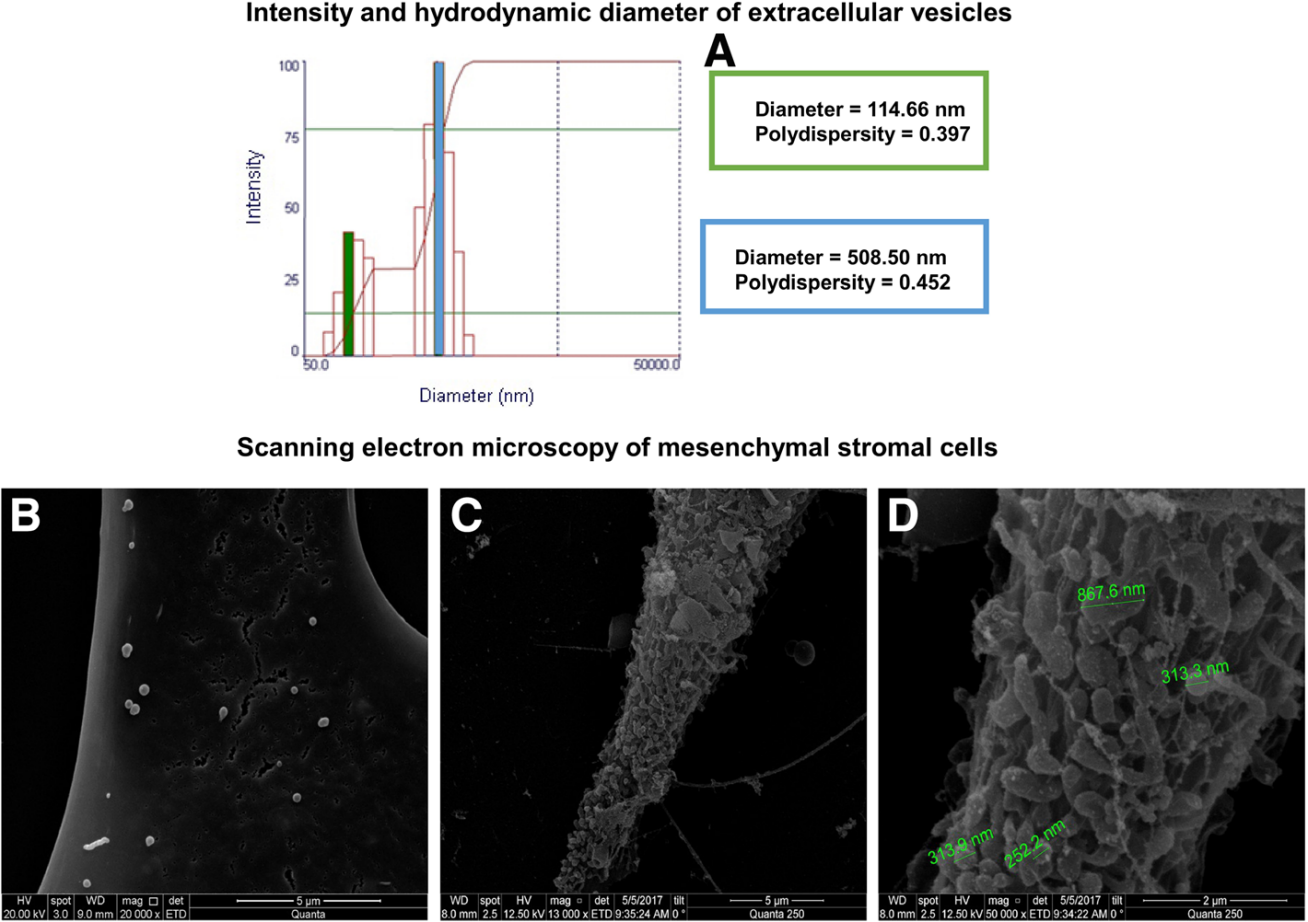
Characterization of EVs. **a** Representative graph of the intensity and hydrodynamic diameter of extracellular vesicle samples analyzed using the dynamic light scattering technique. Graph showing two populations of extracellular vesicles obtained from human adipose tissue-derived mesenchymal stromal cells, one of lower intensity and medium size, characteristic of exosomes, and another with greater intensity and average size, characteristic of microvesicles. **b**, **c**, and **d** Scanning electron microscopy of mesenchymal stromal cells. **b** Mesenchymal stromal cells before induction of cellular stress (fetal bovine serum deprivation), showing the presence of few extracellular vesicles. **c** Image obtained 12 hours after cellular stress induction, showing increase in the number of vesicles in the cell surface. **d** Higher magnification of image C, showing extracellular vesicles with some indicative sizes

The total protein content of the EV fraction was quantified by bicinchoninic acid assay [13]. Intensity and hydrodynamic diameter of EVs were measured by dynamic light scattering (DLS) in a Zetasizer Nano ZS90 system (Malvern Instruments Ltd, Malvern, UK). Twelve hours after the depletion of FBS, AD-MSCs were fixed in 2.5% glutaraldehyde in 0.1 M sodium cacodylate buffer (pH 7.2) for 2 hours and washed twice with cacodylate buffer. Immediately thereafter, post-fixation with OsO_4_ and FeCNK solution (1:1) for 45 minutes was performed, followed by dehydration in a graded ethanol series for 10 minutes at each concentration (30%, 50%, 70%, 90%, 100%, the latter three times). After critical-point drying, the coverslips were analyzed and images were acquired in a FEI QUANTA 250 scanning electron microscope (FEI, Hillsboro, OR, USA). The coverslip with AD-MSCs not subjected to stress was used as a control (Fig. 1).

### OVA-induced airway inflammation and therapeutic protocols

**Fig. 2:**
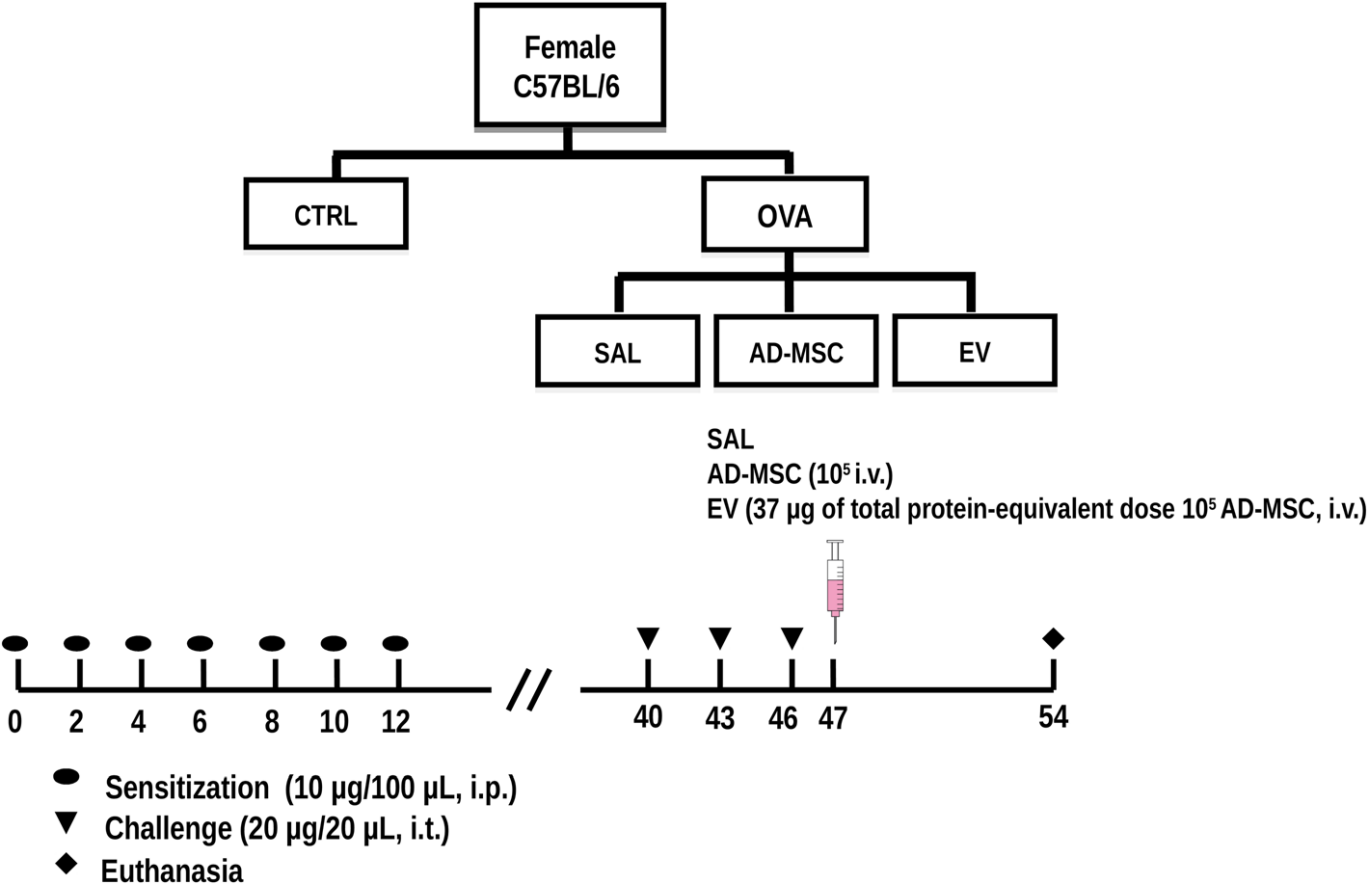
Experimental design. C57BL/6 female mice were divided into two groups: *CTRL* (sensitized and challenged with saline) and *OVA* (sensitized and challenged with ovalbumin). Seven intraperitoneal (i.p.) sensitizations were performed. On days 40, 43, and 46 after first sensitization, an intratracheal challenge (i.t.) was performed. Treatment was administered intravenously (i.v.); 1 day after the last challenge and 7 days after this treatment, the animals were euthanized for data acquisition. The treatment consisted of saline (*SAL*), human adipose-derived mesenchymal stromal cells (*AD-MSC*), or extracellular vesicles (*EV*) at a dose equivalent to 10^5^ AD-MSC (37 μg of total protein)

C57BL/6 mice were randomly divided into two groups. In the ovalbumin group (OVA), mice were immunized using an adjuvant-free protocol by intraperitoneal injection of ovalbumin (100 μg in 100 μL saline) on 7 alternate days. Forty days after the first injection, 20 μg OVA in 20 μL sterile saline were instilled intratracheally. This procedure was performed three times with a 3-day interval between them [14–16]. CTRL animals received saline instead of OVA during the sensitization and challenge steps. In C57BL/6 mice, this protocol induces inflammation and remodeling of central and distal airways as well as lung parenchyma, leading to respiratory mechanical changes [15]. One day after the last challenge, the CTRL and OVA groups were randomized, anesthetized using 5% isoflurane, and treated by jugular injection of 50 μL saline, 10^5^ AD-MSC, or 37 μg EV (equivalent to a 10^5^ dose of AD-MSC) (Fig. 2).

### Lung mechanics

Seven days after AD-MSC or EV administration, mice were sedated with intraperitoneal (i.p.) diazepam (5 mg/kg), anesthetized with sodium thiopental (20 mg/kg i.p.), tracheotomized, paralyzed with vecuronium bromide (0.005 mg/kg) intravenously, and ventilated with a constant-flow ventilator (Samay VR15; Universidad de la Republica, Montevideo, Uruguay). The anterior chest wall was surgically removed and a positive end-expiratory pressure (PEEP) of 2 cm H_2_O was applied. Airflow and tracheal pressure were measured [17] and lung mechanics analyzed by the end-inflation occlusion method [18]. In an open chest preparation, tracheal pressure reflects transpulmonary pressure [19]. After end-inspiratory occlusion, there is an initial rapid decline in transpulmonary pressure (ΔP1,L) from the preocclusion value down to an inflection point, followed by a slow pressure decay (ΔP2,L), until a plateau is reached corresponding to the elastic recoil pressure of the lung. ΔP1,L reflects the pressure used to overcome the airway resistance, while ΔP2,L reflects the pressure produced by stress relaxation, or the viscoelastic properties of the lung. Static lung elastance (Est,L) was determined by dividing the elastic recoil pressure of the lung by the tidal volume. Lung mechanics measurements were performed ten times in each mouse. All data were analyzed in ANADAT software (RHT-InfoData, Inc., Montreal, QC, Canada).

### Lung histology

The left lung was separated from the trachea, esophagus, and heart and fixed in 4% paraformaldehyde solution. Slices (4 μm thick) were cut, deparaffinized, stained with Sirius Red solution, and scanned in a Pannoramic DESK system (3DHistech, Budapest, Hungary). The total number of leukocytes in the peribronchial area was calculated using Case Viewer software (version 1.3; 3DHistech, Budapest, Hungary). The population of eosinophils from each peribronchial region was counted in Pannoramic Viewer software (version 1.15; 3DHistech, Budapest, Hungary). The number of eosinophils was divided by the total number of cells to ascertain the percentage of lung eosinophils [20–22]. For quantification of collagen fibers in the airways and lung parenchyma, slides were stained with Sirius Red dissolved in saturated picric acid for subsequent analysis through polarized-light optical microscopy (BX51, Olympus Latin America Inc., Miami, FL, USA), under × 40 magnification. ImagePro software (version 4.0, Media Cybernetics, Silver Spring, MD, USA) was used to record the areas occupied by collagen, which were then divided by the total area examined [23, 24].

### Enzyme-linked immunosorbent assay (ELISA)

Lung-tissue homogenates were used for cytokine quantification. Briefly, the entire right lung was isolated, homogenized in lysis buffer solution, centrifuged (600 × *g* for 5 minutes and 10,000 × *g* for 10 minutes), and the resulting supernatant was assayed. A sandwich ELISA for interleukin (IL)-10, IL-5, and transforming growth factor beta (TGF-β) (BioLegend, San Diego, CA, USA) was performed as per manufacturer instructions, as were assays for eotaxin, IL-4, IL-13, and interferon (IFN)-γ (Peprotech, Rocky Hill, NJ, USA).

### Total and differential cell counts in bronchoalveolar lavage fluid

Additional female mice were used to analyze total and differential cell counts in BALF (n = 10/group). For this purpose, a polyethylene cannula was inserted into the trachea and a total volume of 1.0 mL of PBS containing 10 mM EDTA was instilled and aspirated (only once). BALF was centrifuged at 300 × *g* for 10 minutes at 4 °C. The supernatant was removed and the pellet resuspended in 250 μL PBS. Total leukocyte counts in BALF were quantitated in Neubauer chambers under light microscopy after dilution of the samples in Türk solution (2% acetic acid).

Cell suspensions from BALF were stained with monoclonal anti-mouse CD3 (Pe-Cy5-labeled) and CD4 (FITC-labeled) antibodies (eBioscience, San Diego, CA, USA) to assess CD3^+^CD4^+^ T-cell percentages. The regulatory T cell (Treg) population was characterized by staining for CD4, CD25, and Foxp3, as per manufacturer instructions (Mouse Treg Staining Kit, eBioscience, San Diego, CA, USA). Additionally, anti-mouse Siglec-F antibody (PE-labeled; BD Pharmingen, San Diego, CA, USA) was used to quantify eosinophils in the polymorphonuclear-gated populations in BALF samples. All data were acquired in a FACSCalibur flow cytometer (BD Biosciences Immunocytometry Systems, San Jose, CA, USA) and analyzed using FlowJo X 10.0.7 software (Tree Star Inc., Ashland, OR, USA).

### Total and differential cell counts in thymus and mediastinal lymph nodes

Thymuses and mediastinal lymph nodes were removed from mice, placed into 1 mL PBS, and homogenized. Total cells in thymus and lymph nodes were counted in a Neubauer chamber after dilution in Türk solution. Cell suspensions from thymus and lymph nodes were stained with monoclonal anti-mouse CD3 (Pe-Cy5-labeled) and CD4 (FITC-labeled) antibodies (eBioscience, San Diego, CA, USA) to assess CD3^+^CD4^+^ T cell percentages. These data were acquired in a FACSCalibur flow cytometer (BD Biosciences Immunocytometry Systems, San Jose, CA, USA) and analyzed using FlowJo X 10.0.7 software (Tree Star Inc., Ashland, OR, USA).

### Statistical analysis

Sample size calculation for testing the primary hypothesis (increased eosinophil counts in BALF in the OVA group) was based on previous measurements [25] and on pilot studies. A sample size of ten animals per group (considering one animal as dropout) was deemed to provide the appropriate power (1-β = 0.8) to identify significant (α = 0.05) differences in eosinophil count between OVA animals and those treated with EVs, with an effect size (d) = 1.9, a two-sided test, and a sample size ratio = 1 (G*Power 3.1.9.2, University of Düsseldorf, Düsseldorf, Germany).

Since lung mechanics and histological analysis were performed in different groups of animals, sample size was calculated on the basis of pilot studies. A sample size of seven animals per group would provide the appropriate power (1 − β = 0.8) to identify significant (α = 0.05) differences in static lung elastance between OVA animals and those treated with EVs, taking into account an effect size d = 1.38, a two-sided test, and a sample size ratio = 1 (G*Power 3.1.9.2, University of Düsseldorf, Düsseldorf, Germany).

Data were tested for normality (Kolmogorov-Smirnov test with Lilliefors’ correction) and homogeneity of variances (Levene median test). Parametric data are expressed as mean ± SD, and nonparametric data as median and interquartile range (IQR). One-way ANOVA with Bonferroni’s post hoc test or Kruskal-Wallis test followed by Dunn’s post-test was used to compare all parameters. *P* values of 0.05 or less were considered significant. All tests were performed in the GraphPad Prism v6.0 statistical software package (GraphPad Software, La Jolla, CA, USA).

## Results

### Respiratory mechanics

**Fig. 3:**
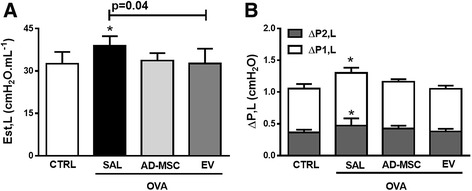
Lung mechanics. **a** Static lung elastance (*Est,L*). **b** Lung resistive (Δ*P1*, *white bar*) and viscoelastic (Δ*P2*, *gray bar*) pressures. Data are presented as means ± SD of seven animals/group. *CTRL* mice sensitized and challenged with saline, *OVA* mice sensitized and challenged with ovalbumin, *OVA-SAL* OVA mice treated with saline, *OVA-AD-MSC* OVA mice treated with mesenchymal stromal cells derived from adipose tissue (AD-MSCs), *OVA-EV* OVA mice treated with extracellular vesicles derived from AD-MSCs. CTRL versus OVA-SAL (Est,L *p* = 0.04, ΔP1, *p* = 0.003, ΔP2, *p* = 0.005)

Est,L, ΔP1,L, and ΔP2,L were significantly higher in OVA-SAL than in CTRL mice (Fig. 3). Systemic administration of EVs only reduced Est,L significantly, while administration of AD-MSCs failed to modify allergen-induced functional changes (Fig. 3).

### Lung remodeling

**Fig. 4:**
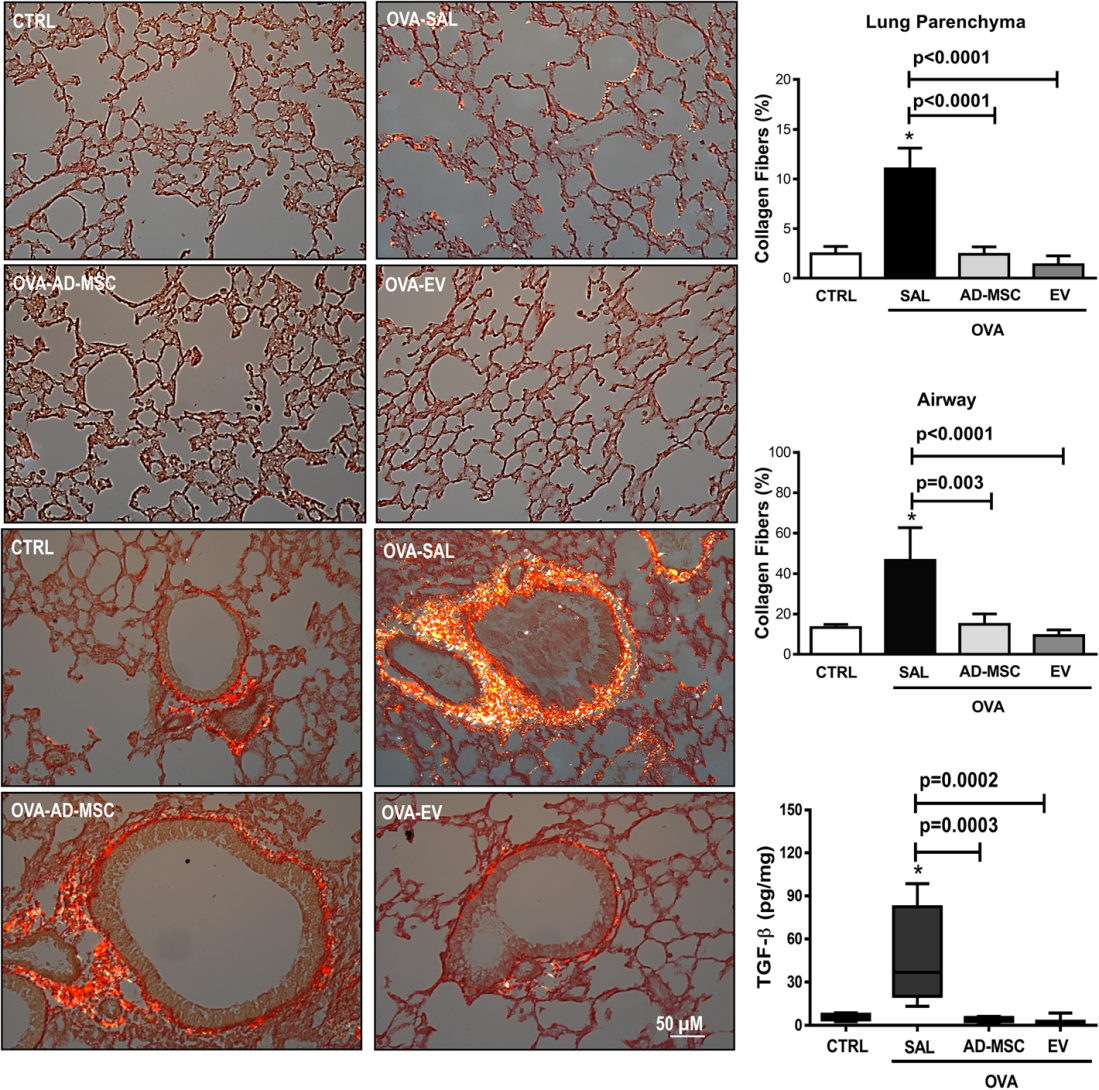
Representative photomicrographs of lung parenchyma (*upper panels*) and airway (*lower panels*) stained with Sirius Red under polarization. Percentage of collagen fiber content in lung parenchyma and airway. Levels of transforming growth factor beta (*TGF-β*) in lung tissue. Data are presented as means + SD of seven animals/group. *CTRL* mice sensitized and challenged with saline, *OVA* mice sensitized and challenged with ovalbumin, *OVA-SAL* OVA mice treated with saline, *OVA-AD-MSC* OVA mice treated with mesenchymal stromal cells derived from adipose tissue (AD-MSCs), *OVA-EV* OVA mice treated with extracellular vesicles derived from AD-MSCs. CTRL versus OVA-SAL (parenchyma *p* < 0.0001, airway *p* = 0.0002, TGF-β *p* = 0.0007)

In OVA-SAL animals, collagen fiber content increased significantly both in lung parenchyma and airways compared to CTRL (Fig. 4). Systemic administration of AD-MSCs and EVs significantly decreased collagen fiber deposition in lung parenchyma and airways (Fig. 4). Lung tissue TGF-β levels were higher in OVA-SAL than CTRL, and reduced significantly both by AD-MSC and by EVs, with no significant differences between these groups.

### Lung inflammation

**Fig. 5:**
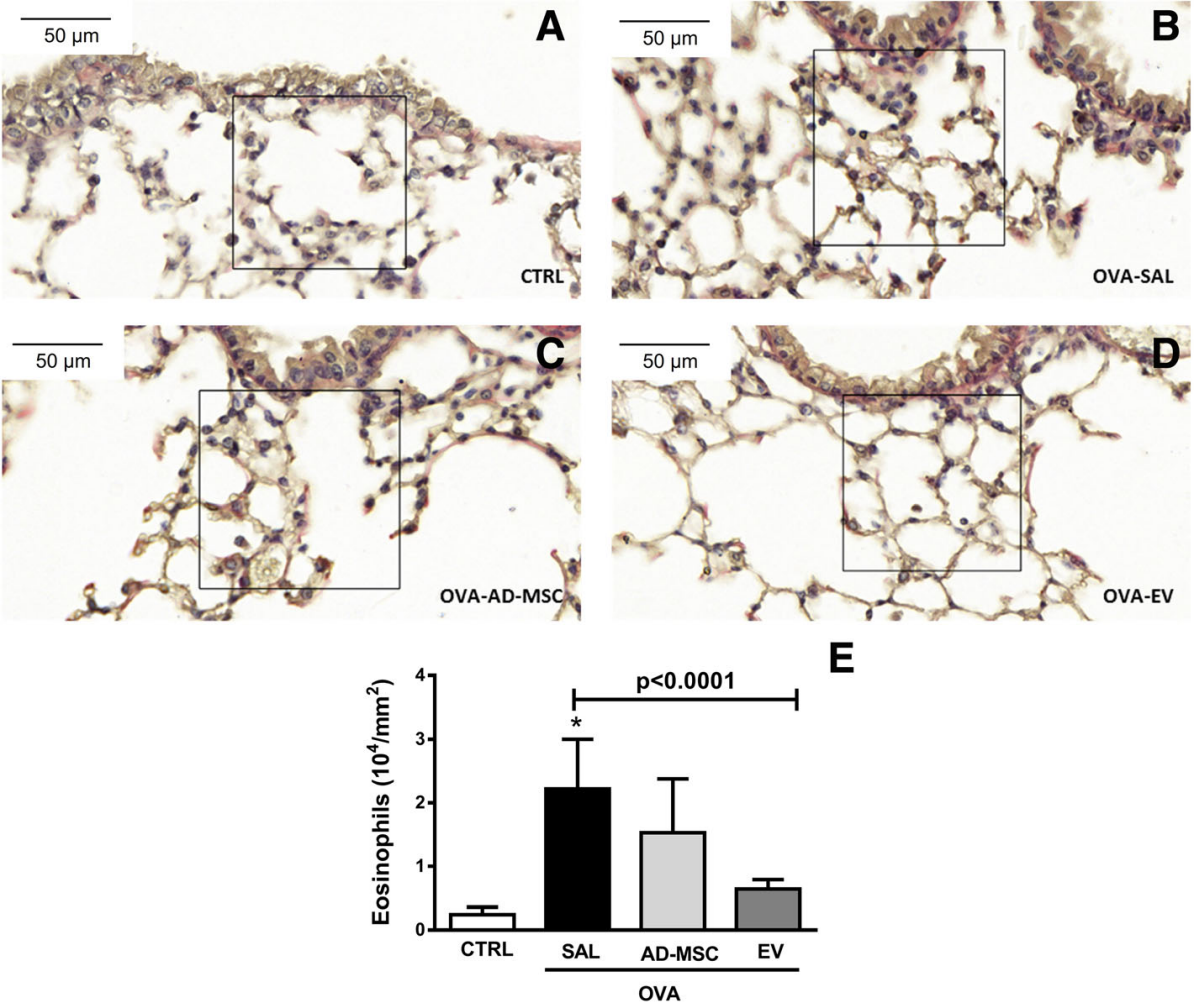
Representative photomicrographs of lung parenchyma stained with Sirius Red. *Squares* indicate peribronchial area. *Lower panel*: number of eosinophils in the peribronchial area. Data are presented as means + SD of seven animals/group. **a** *CTRL* mice sensitized and challenged with saline, **b** *OVA-SAL* *OVA* mice sensitized and challenged with ovalbumin and then treated with saline, **c** *OVA-AD-MSC* OVA mice treated with mesenchymal stromal cells derived from adipose tissue (AD-MSCs), **d** *OVA-EV* OVA mice treated with extracellular vesicles derived from AD-MSCs, **e** Number of eosinophils in lung parenchyma. CTRL versus OVA-SAL (*p* < 0.0001)

**Fig. 6:**
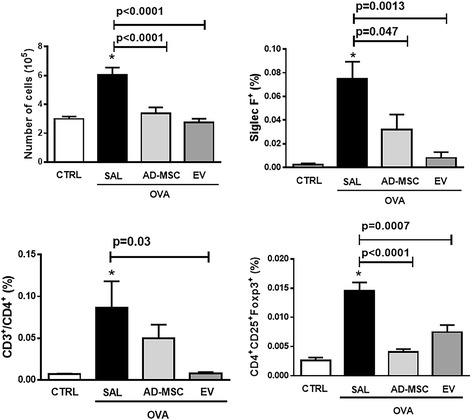
Total cell count and percentage of T cells (CD3^+^CD4^+^), Treg cells (CD4^+^CD25^+^Foxp3^+^), and eosinophils (Siglec-F^+^) in bronchoalveolar lavage fluid (BALF). Data are presented as means + SD of ten animals/group. *CTRL* mice sensitized and challenged with saline, *OVA* mice sensitized and challenged with ovalbumin, *OVA-SAL* OVA mice treated with saline, *OVA-AD-MSC* OVA mice treated with mesenchymal stromal cells derived from adipose tissue (AD-MSCs), *OVA-EV* OVA mice treated with extracellular vesicles derived from AD-MSCs. CTRL versus OVA-SAL (total cell count *p* < 0.0001, eosinophils *p* = 0.0006, Treg cells *p* < 0.0001, T cells *p* = 0.034)

**Fig. 7:**
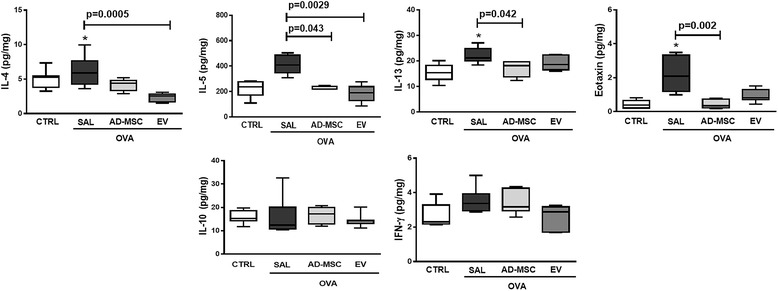
Levels of interleukin (*IL*)-4, IL-5, IL-13, eotaxin, IL-10, and interferon (*IFN*)-γ. Data are presented as median and interquartile range of seven animals/group. *CTRL* mice sensitized and challenged with saline, *OVA* mice sensitized and challenged with ovalbumin, *OVA-SAL* OVA mice treated with saline, *OVA-AD-MSC* OVA mice treated with mesenchymal stromal cells derived from adipose tissue (AD-MSCs), *OVA-EV* OVA mice treated with extracellular vesicles derived from AD-MSCs. CTRL versus OVA-SAL (IL-13 *p* = 0.004, eotaxin *p* = 0.0011)

Systemic administration of EVs, but not AD-MSCs, significantly reduced eosinophil cell counts in lung tissue (Fig. 5). Total cell, eosinophil, CD4^+^CD25^+^Foxp3^+^ regulatory T cell, and CD3^+^CD4^+^ T cell counts in BALF were higher in OVA-SAL than CTRL animals (Fig. 6). Systemic administration of AD-MSCs and EVs significantly reduced BALF total leukocyte and eosinophil counts (Siglec-F), while only AD-MSCs reduced CD4^+^CD25^+^Foxp3^+^ regulatory T cell counts and only EVs reduced CD3^+^CD4^+^ T cell counts (Fig. 6). In the OVA-SAL group, lung tissue levels of pro-inflammatory mediators (IL-4, IL-13, and eotaxin) were higher than in CTRL animals (Fig. 7). Systemic administration of AD-MSCs significantly decreased IL-5, IL-13, and eotaxin levels, while treatment with EVs significantly decreased IL-4 and IL-5. No significant differences were observed in IFN-γ or IL-10 among groups (Fig. 7).

### Inflammatory cells in primary and secondary lymphoid tissues

**Fig. 8:**
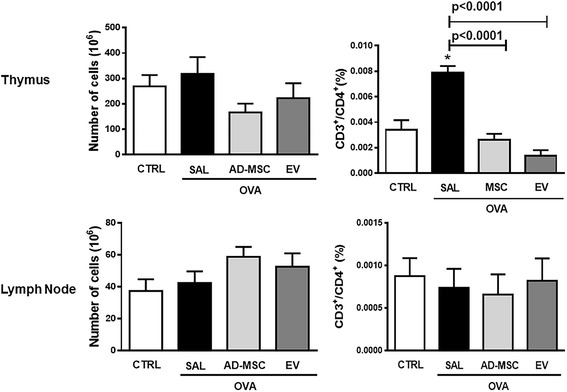
Total cell count and percentage of T cells (CD3^+^CD4^+^) counts in thymus and mediastinal lymph nodes. Data are presented as means + SD of ten animals/group. *CTRL* mice sensitized and challenged with saline, *OVA* mice sensitized and challenged with ovalbumin, *OVA-SAL* OVA mice treated with saline, *OVA-AD-MSC* OVA mice treated with mesenchymal stromal cells derived from adipose tissue (AD-MSCs), *OVA-EV* OVA mice treated with extracellular vesicles derived from AD-MSCs. CTRL versus OVA-SAL (thymus *p* = 0.0002)

In the thymus, the total number of CD3^+^CD4^+^ T cells was increased in OVA-SAL compared to CTRL animals, and decreased significantly after systemic administration of AD-MSCs or EVs (Fig. 8). No significant changes in total cell count or percentage of CD3^+^CD4^+^ T cells in lymph nodes were observed across groups (Fig. 8).

## Discussion

In the present study, both AD-MSCs and EVs similarly reduced collagen fiber deposition in the lung parenchyma and airways, TGF-β levels in lung tissue, total cell counts, CD4^+^CD25^+^Foxp3^+^ (Treg cells) and eosinophil percentage in BALF, IL-5 levels in lung tissue, and percentage of CD3^+^CD4^+^ T cells in the thymus. In contrast, systemic administration of AD-MSCs or EVs had different effects on eosinophil cell counts, levels of IL-4, IL-13, and eotaxin in lung tissue and CD3^+^CD4^+^ T cells in BALF, and lung mechanics (Table 1).

**Table 1:** Comparison between human adipose-derived mesenchymal stromal cells (AD-MSC) with extracellular vesicles (EV) in OVA-induced allergic asthma model

Parameters	AD-MSC	EV
Reduction of static lung elastance	✕	✓
Reduction of collagen fiber content in lung parenchyma and airway	✓	✓
Reduction of eosinophils in in lung parenchyma	✕	✓
Reduction of T lymphocytes in BALF	✕	✓
Increase of Treg cells in BALF	✕	✕
Reduction of eosinophils in BALF	✓	✓
Reduction of TGF-β in lung tissue	✓	✓
Reduction of eotaxin in lung tissue	✓	✓
Reduction of IL-5 in lung tissue	✓	✓
Reduction of IL-4 in lung tissue	✕	✓
Reduction of IL-13 in lung tissue	✓	✕

X means inability to promote parameter improvement and ✓ means ability to promote the parameter improvement

*BALF* bronchoalveolar lavage fluid, *Treg* regulatory T cells, *TGF* transforming growth factor, *IL* interleukin

The model of allergic asthma used herein in was developed by our group [15] and has been previously demonstrated to induce changes in lung mechanics, inflammation, and airway remodeling (mucous-cell hyperplasia, presence of myofibroblasts, subepithelial fibrosis and thickening of the basement membrane, smooth muscle cell hypertrophy, and deposition of collagen fibers in the airways and lung parenchyma) [15, 16]. Female C57BL/6 mice were used as they mount a good allergic response to ovalbumin [15, 16, 26] and are more susceptible to experimental asthma than males [26].

AD-MSCs are easily obtained in large numbers, present high proliferation rates compared to bone marrow-MSCs [27], and have shown beneficial effects when administered systemically before sensitization or during challenge in OVA-induced asthma models [6–9]. However, to date, no study had evaluated the therapeutic administration of human AD-MSCs and their EVs in experimental allergic asthma, when airway remodeling is already established. Both AD-MSCs and EVs were administered intravenously, as a previous study found no differences in outcomes after intratracheal versus *i*ntravenous administration of bone marrow-derived MSCs in experimental asthma [15]. Additionally, recent studies have shown that a systemic dose of 10^5^ BM-MSC per mouse, similar to that used in this study, is effective in experimental asthma [15, 28]. In addition to this dose of AD-MSCs, we used an equivalent dose of EVs secreted by 10^5^ AD-MSCs, since recent studies reported that human MSC-derived EVs were as effective as their parent stem cells in mitigating lung inflammation in experimental lung injury [11, 29, 30]. Based on our previous studies, both AD-MSCs and EVs were administered 24 hours after the last challenge, when airway inflammation and remodeling processes are established and lung function is already compromised [23, 24], thus resembling human asthma.

We observed that EVs (but not human AD-MSCs) reduced static lung elastance, in contrast with other studies that reported decreased lung mechanical changes after administration of human AD-MSCs [6, 8, 10]. These differences could be attributed to several factors: (1) the number of AD-MSCs administered, (2) the timing of AD-MSCs systemic administration (before or after allergic asthma induction), (3) the severity of asthma, and (4) the method used to measure lung mechanical parameters. In the present study, in contrast to the literature, human AD-MSCs were administered both in lower numbers and after airway remodeling was already established. Moreover, lung mechanics were measured by end-inflation method, but not by flexiVent or the Penh system.

The model of OVA-induced allergic asthma used in this study is characterized by T helper 2 cell release of cytokines such as IL-4, IL-5, and IL-13, as well as the eosinophil chemoattractant eotaxin, which contribute to airway inflammation in asthma [23, 24] Among other functions, these cytokines induce fibroblast proliferation, extracellular matrix deposition, airway hyperresponsiveness, epithelial cell apoptosis, mucus production, and eosinophil recruitment [31]. Therefore, they play important roles not only in the inflammatory process, but also in airway remodeling, and are thus considered important therapeutic targets [32, 33]. AD-MSCs decreased IL-5, IL-13, and eotaxin levels in lung tissue, perhaps by decreasing the number of eosinophils in BALF [33, 34]. TGF-β also plays an important role in tissue remodeling. TGF-β is produced by epithelial cells, fibroblasts, eosinophils, and macrophages, and stimulates the production of collagen I and III, fibronectin, proteoglycans (fibroblasts) [35–37]. Both EVs and AD-MSCs similarly reduced collagen fiber content and TGF-β levels. Our results suggest that AD-MSCs and EVs seem to modulate inflammation through different mechanisms, but had similar effects on the remodeling process.

IFN-γ produced predominantly by Th1 cells can counterbalance the allergic inflammatory response mediated by Th2 cells, and it is present in BALF of asthmatic humans and mice [38]. IL-10 can be released by many cell types, such as Th2 cells, Treg cells, mast cells, eosinophils, and macrophages [39]; in this study, no significant changes were observed between groups despite the increased number of eosinophils and Treg cells in BALF. IFN-γ and IL-10 levels did not increase after ovalbumin-induced allergic asthma in accordance with previous studies [25, 40]. The absence of changes in IFN-γ and IL-10 may be related to the timing of analysis (7 days after the last challenge), since such modifications have been described during asthma resolution [41].

Human bone marrow-derived MSCs injected in mice migrate to the thymus, inhibiting maturation of naive lymphocytes into CD3^+^CD4^+^ T cells [42, 43]. In our studies, no changes were observed in the CD3^+^CD4^+^ T cell populations in mediastinal lymph node tissue after induction of asthma with ovalbumin. Both AD-MSCs and EVs decreased the number of CD3^+^CD4^+^ T cells in the thymus, but only systemic administration of EVs caused a reduction of CD3^+^CD4^+^ T cells in BALF. Both AD-MSCs and EVs reduced CD3^+^CD4^+^ T cells in the thymus, but only EVs inhibited accumulation of CD3^+^CD4^+^ T cells in the lung. One mechanism of MSC action may be through induction of Treg cells [44]. MSCs derived from mouse adipose tissue induce Treg cells in an ovalbumin-induced asthma model [6]; however, Luz-Crawford et al. [45] demonstrated that MSCs derived from mouse bone marrow modulate Treg cell levels only when the MSCs are co-cultured with lymphocytes before the differentiation process, not when the lymphocytes are already differentiated. In experimental asthma, after sensitization and challenge with *Aspergillus* hyphae, Treg cells were increased in the spleen and not modified after systemic administration of MSCs [46].

Similarly, there were no differences in Treg cell counts in the mediastinal lymph nodes across the groups analyzed in the present study. Treg cell counts in BALF increased in OVA animals compared to control animals, which may be associated to the timing of analysis, i.e., late in the course of asthma, when both inflammation and remodeling are clearly established. Systemic administration of AD-MSCs and EVs reduced levels of Treg cells in BALF.

The current study has some limitations that should be addressed. First, neither AD-MSCs nor EVs were tracked after systemic administration, limiting our knowledge concerning their delivery and homing. Second, only one dose of human AD-MSCs and EVs was evaluated; thus, we cannot rule out that higher doses may result in greater beneficial effects. Certainly, further studies performing dose-response curves of AD-MSCs and EVs are required. In this model of allergic asthma, airway inflammation and remodeling persists for some time after the challenges [17], which may allow ascertainment of whether the beneficial effects observed remain. This experimental study is a first step toward other investigations to elucidate the mechanisms and time course of therapy with AD-MSCs and EVs in asthma. Finally, a specific set of cytokines and growth factors was evaluated in this experiment; a wider range of mediators should be analyzed to further elucidate the mechanisms of action of AD-MSCs and EVs.

## Conclusions

Administered systemically, both AD-MSCs and their EVs had beneficial effects in the model of ovalbumin-induced allergic asthma used herein, acting on the inflammatory process (reduction in total cell counts and eosinophil percentage in BALF, IL-5 levels in lung tissue, and percentage of CD3^+^CD4^+^ T cells in the thymus) and reversing tissue remodeling (decreased collagen fiber deposition in the lung parenchyma and airways, reduced TGF-β levels in lung tissue). While the effects of each were largely similar, differences were observed in outcome assessment of lung mechanics and inflammation. In this line, systemic administration of AD-MSCs or EVs had different effects on eosinophil cell counts, levels of IL-4, IL-13, and eotaxin in lung tissue, CD3^+^CD4^+^ T cells in BALF, and lung mechanics. This should encourage further study into the differential mechanisms by which MSCs versus EVs might act in respiratory disease.

## Abbreviations

AD-MSC: Human adipose tissue-derived mesenchymal stromal cell
BALF: Bronchoalveolar lavage fluid
DMEM: Dulbecco’s modified Eagle’s medium
ELISA: Enzyme-linked immunosorbent assay
EV: Extracellular vesicle
FBS: Fetal bovine serum
IFN: Interferon
IL: Interleukin
OVA: Ovalbumin
PBS: Phosphate-buffered saline
TGF: Transforming growth factor
Treg: Regulatory T cells
